# Aberrant allele-specific replication, independent of parental origin, in blood cells of cancer patients

**DOI:** 10.1186/1471-2407-8-390

**Published:** 2008-12-25

**Authors:** Zohar A Dotan, Aviva Dotan, Jacob Ramon, Lydia Avivi

**Affiliations:** 1 Department of Urology, Sheba Medical Center, Tel-Hashomer 52621, Israel; 2 Department of Human Molecular Genetics and Biochemistry, Sackler School of Medicine, Tel Aviv University, Tel Aviv 69978, Israel

## Abstract

**Background:**

Allelic counterparts of biallelically expressed genes display an epigenetic symmetry normally manifested by synchronous replication, different from genes subjected to monoallelic expression, which normally are characterized by an asynchronous mode of replication (well exemplified by the *SNRPN *imprinted locus). Malignancy was documented to be associated with gross modifications in the inherent replication-timing coordination between allelic counterparts of imprinted genes as well as of biallelically expressed loci. The cancer-related allelic replication timing aberrations are non-disease specific and appear in peripheral blood cells of cancer patients, including those with solid tumors. As such they offer potential blood markers for non-invasive cancer test. The present study was aimed to gain some insight into the mechanism leading to the replication timing alterations of genes in blood lymphocytes of cancer patients.

**Methods:**

Peripheral blood samples derived from patients with prostate cancer were chosen to represent the cancerous status, and samples taken from patients with no cancer but with benign prostate hyperplasia were used to portray the normal status. Fluorescence *In Situ *Hybridization (FISH) replication assay, applied to phytohemagglutinin (PHA)-stimulated blood lymphocytes, was used to evaluate the temporal order (either synchronous or asynchronous) of genes in the patients' cells.

**Results:**

We demonstrated that: (i) the aberrant epigenetic profile, as delineated by the cancer status, is a reversible modification, evidenced by our ability to restore the normal patterns of replication in three unrelated loci (*CEN15*, *SNRPN *and *RB1*) by introducing an archetypical demethylating agent, 5-azacytidine; (ii) following the rehabilitating effect of demethylation, an imprinted gene (*SNRPN*) retains its original parental imprint; and (iii) the choice of an allele between early or late replication in the aberrant asynchronous replication, delineated by the cancer status, is not random but is independent of the parental origin.

**Conclusion:**

The non-disease specific aberrant epigenetic profile displayed in peripheral blood cells of patients with a solid tumour (unlike genetic aberrations) can be reversed, by an epigenetic drug applied in vitro, to the normal. It appears that the cancerous status differentiates between two allelic counterparts in a non-random manner, but independent of the parental origin

## Background

The fundamentals of Mendelian genetics lead to the assumption that genes of the parental genomes in mammals keep a functional symmetry: the two alleles function or shut off concomitantly in what is called biallelic expression. However, a subset of the genes is subjected to allele-specific expression (monoallelic expression), in which only one allele retains expression capability while its counterpart is silent [1,2]. Monoallelically expressed genes include imprinted genes [3-5], X-linked genes subjected in female cells to X-chromosome inactivation [6,7], and genes displaying allelic exclusion [8,9].

The functional asymmetry of alleles of an imprinted gene depends upon the parental origin of the allele – whether maternal or paternal. It is established during germ-cell development into sperm or eggs, and after fertilization each allele maintains its parental imprint, which segregates almost unchanged in the developing organism [5].

In contrast to the process of imprinting, in the processes of X-inactivation and allelic exclusion, the choice of an allele to be activated or silenced is not associated with parental origin. In the X-inactivation and allelic exclusion processes the functional capability or incapability is determined in a kind of stochastic selection by an as yet unknown selection in each individual cell: one allele stays potentially active and its partner becomes incapable of expressing itself [reviewed in [10]]. This pattern is normally maintained in a clonally-dependent manner throughout cell proliferation, enabling each tissue to carry a potentially active paternal allele in some cells and a potentially active maternal allele in other cells, the frequencies of the two cell types usually deviate from random; in some cases most of the cells (of a given tissue) carry an active maternal allele while in others most of the cells (of the very same tissue) display an active paternal allele, giving rise to a non-random pattern but independent of the parental origin [6,7,11-14].

Whatever the mechanisms involved in the maintenance and selection of an allele for allele-specific expression, the functional asymmetry of monoallelically expressed genes results from the two alleles maintaining different epigenetic profiles, in which asynchronous DNA replication, similar to differential DNA methylation, plays a decisive role [reviewed in [12]].

The method of choice for evaluating the temporal order of allelic replication is the fluorescence in situ hybridization (FISH) replication assay [11,15-18]. This assay was first developed to confirm and reinstate previous observations that two homologous counterparts usually replicate concomitantly, and to demonstrate unequivocally that the two alleles of a biallelically expressed gene replicate synchronously, early in cells of expression and late in unexpressed cells [19]. Using this assay an asynchronous pattern of allelic replication – early replication of the potentially active allele and late replication of the silent one – was shown, not necessarily in the cells of expression, for all known types of monoallelically expressed genes: (i) imprinted genes [20-26], (ii) genes subjected to X-chromosome inactivation [27-29], and (iii) genes undergoing allelic exclusion [11,13,15,30,31].

We reported previously that imprinted genes lose their characteristic epigenetic-asymmetry, as reflected in loss of asynchronous replication, in peripheral blood lymphocytes of patients with a solid tumor such as renal cell carcinoma [25] or prostate cancer [26]. This is in accord with studies documenting loss of the allele-differential methylation characterizing imprinted genes, a phenomenon often referred to as "loss of imprinting" (LOI) in peripheral blood lymphocytes of patients with a solid tumor such as colon cancer [32,33]. Furthermore, classical biallelically expressed genes, exemplified by *RB1*, *TP53*, *AML1 *and *C-MYC*, which normally display a synchronous mode of allelic replication, yet in peripheral blood lymphocytes of patients with a solid tumor, such as renal cell carcinoma [25] or prostate cancer [26], exhibit an asynchronous pattern of replication similar to that characterizing normally monoallelically expressed genes. Even satellite chromosome-specific sequences (pericentromeric non-coding DNA arrays), which normally display synchrony in replication of homologous counterparts, similar to biallelically expressed genes, change their inherent replication mode and replicate asynchronously in lymphocytes of patients with cancer, including ovarian [34], hematological [35] and prostate cancers [26]. Considering that biallelically expressed genes in cells of cancer patients, similar to imprinted genes, are subjected to the global epigenetic disequilibrium associated with tumorigenesis [36], these replication alterations are inevitable. The feasibility to observe a cancer-linked marker in peripheral blood cells of cancer patients would be of immense value in cancer diagnosis and therapy [37], especially if it is based on DNA itself rather than on a DNA product.

The present study was aimed to gain some insight into the process leading to the replication timing alterations of genes in the blood lymphocytes of cancer patients. Specifically, we checked: (i) if the cancer-related loss of asynchronous replication of an imprinted gene, and "gain" of asynchrony of loci that normally replicate synchronously are linked to aberrant methylation; and (ii) whether the choice of an allele for early or late replication in cancer-related asynchronous replication is random, dependent of the parental origin or non-random but independent of the parental origin.

## Methods

### Study subjects

Forty-four male urology patients were included in the study, 22 with prostate cancer (CAP), and 22 with no cancer but with benign prostate hyperplasia (BPH). Diagnoses were based on histological examination of tissues removed from the prostate gland by surgery or biopsy. The study also included two healthy family members, the wife (designated K1) and son (designated K2) of one of the non-cancer (BPH) patients (designated K). The 44 patients and the two family members of patient K had normal karyotypes, based on G-banded metaphase spreads.

### Lymphocyte cultures

Each subject donated 5 ml of peripheral blood prior to an invasive diagnostic procedure (surgery or biopsy), or medical treatment (hormonal, radiation, or chemotherapy). Cell cultures of PHA-stimulated lymphocytes were set up according to the standard protocol used for routine karyotype assays described previously [26]. Briefly, blood samples were cultured in F10 medium containing 20% fetal calf serum (FCS), 3% phytohemagglutinin (PHA), 0.2% heparin, and 1% antibiotics. Six of the 22 samples derived from the cancer (CAP) patients (designated C1–C6) and six of the 22 samples obtained from the non-cancer (BPH) patients (designated B1–B6) were also cultured in the presence of 10^-7 ^M 5-azacytidine (AZA; Sigma Chemicals, St. Louis, MO. USA), added to the other ingredients of the medium. Cultures were incubated at 37°C for 72 h, colchicine (final concentration of 0.1 μg/ml) was added for 1 h, and hypotonic treatment (0.075 M KCl at 37°C for 15 min) and four washes were carried out, each with a fresh cold fixation solution (3:1 methanol: acetic acid solution). The cell suspensions in the fixative solution were stored at -20°C until analysis by FISH.

### DNA probes

**Figure 1:**
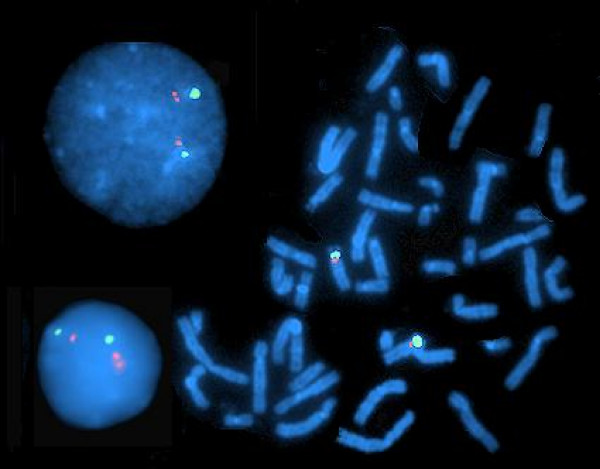
**Cells of a healthy (cancer free) individual (case K2) heterozygous for the large *CEN15 *marker (derived from the paternal parent) after two-color FISH with *SNRPN *(red signals) and *CEN15 *(green signals)**. Left, two interphase cells representing SD cells for *SNRPN *and SS cells for *CEN15*. Right, cell at metaphase with chromosome-15 homologues identified by the *CEN15 *and *SNRPN *signals. Note the difference in signal size between the two *CEN15 *counterparts in each cell, and the association of the replicated *SNRPN *locus (doublet; D-signal) in both interphase cells with the large *CEN15*.

Three directly labeled commercial probes obtained from Vysis Inc. (Downers Grove, IL, USA) were used: (i) the *SNRPN *probe (32–190004), which identifies the imprinted *SNRPN *gene, located on the long arm of chromosome 15 (15q11-q13, within the Prader-Willi/Angelman syndrome imprinted region) adjacent to the centromere; (ii) the *RB1 *Probe (32–190001), which identifies the retinoblastoma tumor suppressor gene, a classical biallelically expressed gene [38]; and (iii) the *CEN15 *probe (32–130015), which identifies the pericentromeric region of chromosome 15 *(CEN15*). *CEN15 *was considered appropriate because it faithfully mimics the replication mode of a biallelically expressed locus, it has a size polymorphism that enables identification of each specific allelic counterpart in informative cells, and it is located in the vicinity of the *SNRPN*, whose replication status tells the parental origin of the chromosome [20,23], enabling easy determination of the parental origin of each *CEN15 *morph (for details see Fig. 1).

### Probe application

Applying one-color FISH, each of the 44 samples derived from the urology patients was hybridized individually with the *SNRPN *probe and the *CEN15 *probe. In addition, each of six samples from the BPH group of patients (cases B1–B6) and each of six samples from the CAP group (cases C1–C6), was hybridized with the *RB1 *probe too.

Of the 44 samples, 11 were polymorphic for a large *CEN15 *marker: five from the BPH group (including patient K) and six from the CAP group. These 11 samples and the sample from the healthy son (K2) of patient K were all hybridized concomitantly with two probes, *SNRPN *and *CEN15 *(two-color FISH). The sample of the wife of patient K (K1) was hybridized with the *CEN15 *probe only and displayed two normal, equal-sized *CEN15 *signals, verifying that the large *CEN15 *marker of K2 (Fig. 1) was of paternal origin.

### *In-situ *hybridization

We followed a protocol previously described [26]. Cells were dropped onto two-well slides (Insitus Biotechnologies, Albuquerque, NM, USA) with no pretreatment. Five μl of the probe solution, diluted in D003 (*SNRPN *and *RB1*) or D001 (*CEN15*) Ingen's DenHyb hybridization solution (Insitus Biotechnologies), were placed on the targeted area of the sample slides, covered with a 12 mm round silianized coverslip (Insitus Biotechnologies), and sealed with rubber cement. The slides were placed in a micro-heating system (Vysis Inc.), programmed first for 6 min denaturation at 76°C and then for 18 hours hybridization at 37°C.

### Post-hybridization treatments

Following removal of the coverslips, post-hybridization washes consisted of immersing the slides for 2 min in a solution of 0.4 × SSC pH 7.0 with 0.3% NP40 at 72°C, followed by 2 min in 2 × SSC with 0.1% NP40 at room temperature in a shaking water bath. After brief drying, the slides were covered with an antifade solution containing 4,6-diamidino-2-phenylindole (DAPI; 3 μg/ml; Vector Laboratories, Inc., Burlingame, CA, USA), coated with glass coverslips, and stored at -20°C until analyzed.

### Cytogenetic evaluation

Slides were analyzed blindly on an Olympus BH2 fluorescent microscope equipped with a triple band-pass filter (Chroma Technology, Brattleboro, VT, USA). The FISH replication assay was used to estimate the replication status of each allelic region as described previously [26]. Accordingly, the structure of the fluorescent signal of each identified region was noted: either singlet (S), denoting a non-replicated sequence, or doublet (D), disclosing a replicated sequence. Thus, in a population of replicating cells, the frequency of cells exhibiting an "S" signal for one allele and a "D" signal for the other (SD cells), out of the total population of cells with two fluorescent signals, represented the level of asynchrony in the replication timing of the identified alleles. High SD-cell frequency indicated an asynchronous mode of replication, and low frequency a synchronous mode [16,17,20].

For estimation of the frequency of SD cells at least 100 cells with two clear signals were scored from each sample, for each treatment, for each locus, following one-color FISH with the relevant probe. To differentiate a specific *CEN15 *or *SNRPN *allele from its allelic counterpart based on the size-polymorphism of the *CEN15 *fluorescent signal, at least 200 SD cells for the locus in question were scored from each informative sample; for *CEN15 *differentiation one-color FISH (with the *CEN15 *probe) and for *SNRPN *differentiation two-color FISH (with the *CEN15 *and the *SNRPN *probes) were applied.

### Statistical method

The statistical significance of the differences between two cell populations was determined using the two-tailed Student's t-test (Microsoft Excel) and a P value < 0.01 was considered statistical significant.

### Ethical basis

Informed consent was obtained from each individual examined, and the Ethics Committee of the Sheba Medical Center approved the study.

## Results

### Replication timing alterations of *SNRPN *and *CEN15 *in cells of cancer patients are readjusted in the presence of 5-azacytidine (AZA, a methylation-blocking agent)

**Figure 2:**
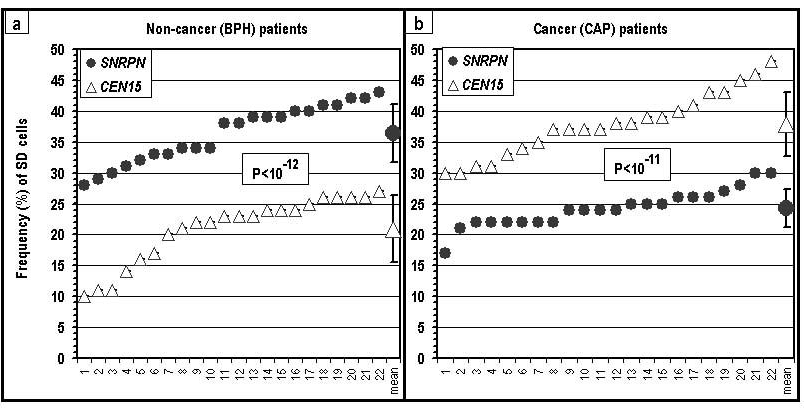
**SD values for *SNRPN *and *CEN15 *in cells of two groups of urology patients**: (a) patients free of cancer (BPH); and (b) cancer patients (CAP). The values in each frame for each locus are presented in increasing order. P – the level of significance of the differences between the *SNRPN *and the *CEN15 *loci within a group of patients.

The frequency of SD cells (see 'Cytogenetic evaluation' in Materials and Methods) for *SNRPN *was high in cells of the cancer-free urology patients, as expected for an imprinted region in normal samples. However, this very same locus in the samples of the cancer patients revealed low SD values, significantly lower (P < 10^-11^) than the expected values (Fig. 2; Table 1). In addition, the *CEN15 *alleles, which normally replicate synchronously, as seen here in the cells of the non-cancer patients (Fig. 2a), replicated highly asynchronously in the cells of the cancer patients, similar (P > 0.30) to the *SNRPN *in the cells of cancer-free patients (Fig. 2; Table 1). Thus, the cells of the cancer patients exhibited an abnormal mode of allelic replication: low SD values for *SNRPN*, which were significantly lower (P < 10^-11^) than the SD values obtained in the same samples for the *CEN15 *locus (Fig. 2b), and only slightly higher (P < 0.02) than the low SD value shown for *CEN15 *in the non-cancer samples (Fig. 2; Table 1). At the same time, the cells of the cancer-free urology patients exhibited the normal pattern of replication: high SD values for *SNRPN *and significantly lower (10^-12^) values for *CEN15 *(Fig. 2a).

**Table 1:** Level of significance of the differences (P) in SD values for the designated loci between samples of patients free of cancer (BPH) and cancer patients (CAP).

	BPH *SNRPN* (36.4 ± 4.7%)	BPH *CEN15* (20.9 ± 5.3%)
CAP *SNRPN* (24.3 ± 3.0%)	P < 10^-11^	P < 0.02

CAP *CEN15* (37.8 ± 5.1%)	P > 0.30	P < 10^-12^

In parentheses are the mean SD frequency (%) and the standard deviation value for the group of patients for the particular locus. Each group contained 22 cases (the compared values are presented in Figure 2).

**Figure 3:**
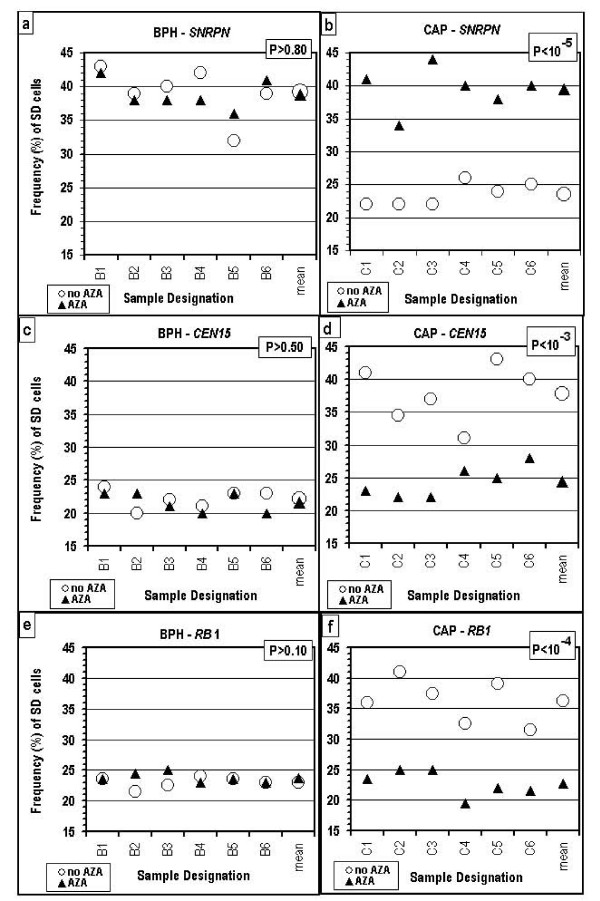
**SD values for the *SNRPN*, *CEN15 *and *RB1 *loci, in the absence (○) and in the presence (▲) of AZA**. (a), (c) and (e) values of patients free of cancer (BPH cases); (b), (d) and (f) values of urology cancer patients (CAP cases). P – the level of significance of the differences between corresponding values obtained in the presence and absence of AZA.

The abnormal SD values for *SNRPN *and *CEN15 *in the cells of the cancer patients were shifted toward normal in the presence of AZA, a methylation-blocking agent (Fig. 3b and 3d). In contrast, the samples of the non-cancer patients were not affected by AZA (Fig. 3a and 3c), evidenced by the lack of difference, in the presence of AZA, between the cancer cases and the non-cancer cases in the SD values for *SNRPN *as well as for *CEN15 *(Fig. 3).

It is noteworthy that *CEN15 *mimics the replication timing of *RB1*: (i) low SD values in cells of cancer-free patients, both with and without AZA; (ii) high SD values in the cells of the cancer patients, similar to those obtained for an imprinted region in normal cells; and (iii) a decrease in the abnormal (high) SD values in the cells of the cancer patients to normal low levels in the presence of AZA (Fig. 3c–f).

### The loss of the asynchronous pattern of *SNRPN *replication in cells of cancer patients is not accompanied by randomization of the early and late replicating alleles

Following one-color FISH with the *CEN15 *probe, 11 of the 44 samples studied were heterozygous for *CEN15 *size: six derived from cancer (CAP) patients (designated Y, J, A, C, B, and S), and five from non-cancer (BPH) patients (designated G, V, R, Z, and K). Each displayed at both interphase and metaphase one large and one small *CEN15 *signal (illustrated in Fig. 1). The son of patient K was a young healthy man (K2) who happened to be heterozygous for a large *CEN15 *marker (Fig. 1), similar to his father (K); his mother (K1) was homozygous for two small *CEN15 *markers (data not shown).

**Figure 4:**
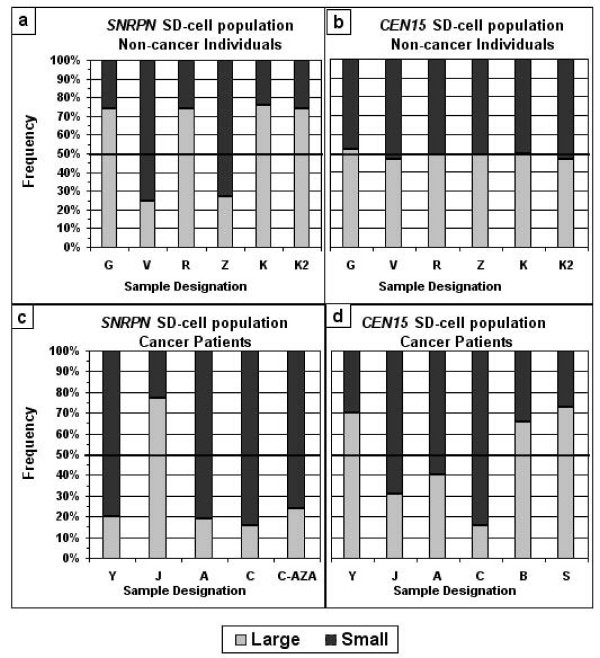
**Characterization of the SD-cell populations for *SNRPN *and *CEN15 *in cell samples of individuals heterozygous for the *CEN15 *size, showing one large and one small *CEN15 *marker**. Light bars show the portion (%) of the relevant SD-cell population in which the replicated region (D-signal) is associated with the large *CEN15 *marker, and dark bars show the SD-cell portion in which the D-signal is associated with the small *CEN15 *marker. (a) and (b) samples of cases free of cancer (K2 was from a healthy young man, the son of K); (c) and (d) samples of cancer patients; the last bar in frame (c) is a sample of cancer patient C (case C5 in Fig. 3) grown in the presence of AZA; at least 200 SD cells were scored from each sample for each assay.

Using two-color FISH to identify the *CEN15 *and the *SNRPN *in the same cell, we examined the population of SD cells for *SNRPN *in the samples heterozygous for the large *CEN15 *marker. The early replicating *SNRPN *allele was not randomly distributed between the two chromosome-15s in either the informative (heterozygous) samples derived from non-cancer cases (Fig. 4a) or those derived from the informative cancer cases (Fig. 4c). Specifically, in the samples of the non-cancer BPH cases (G, R and K), in the K2 sample, and in one sample of a cancer (CAP) case (J), the early replicating *SNRPN *allele was located in more than 70% of SD cells on the chromosome carrying a large *CEN15 *marker. On the other hand, in samples V and Z derived from non-cancer patients, as well as samples Y, A, and C derived from cancer patients, the early replicating *SNRPN *allele was found in more than 75% of SD cells on the chromosome identified by a small *CEN15 *marker (Fig. 4a and 4c). This clearly showed that the relaxation in the asynchronous replication of *SNRPN *(reduced SD-cell frequency) that characterizes samples of cancer patients was not accompanied by randomization of the early and late replicating alleles.

Moreover, the rehabilitating effect of AZA, which restored the normal high *SNRPN *SD values in cancer samples (Fig. 3b), reinstated the early replicating *SNRPN *allele on the same chromosomal counterpart at which the early replicating allele was located in the absence of the drug (apparently the paternal chromosome). This was demonstrated by cancer case C: in most of the SD cell population for *SNRPN *the early replicating allele was on the chromosome identified by a small *CEN15 *marker both before and after AZA application (see the two last bars in Fig. 4c).

Worthy of notice that K2 is of particular interest here because the parental origin (paternal) of the large *CEN15 *marker was known, and the early replicating *SNRPN *allele was indeed associated with the paternal chromosome (see last two bars in Fig. 4a). Moreover, the results of the father (K) and the son (K2) confirmed previous findings [20,23] that, in informative *CEN15 *cases, the replication status of the *SNRPN *locus enables identification of parental origin of chromosome 15 with no need for family studies, which are usually complicated, especially in elderly subjects.

### The allelic choice for an asynchronous pattern of replication for *CEN15 *in cells of cancer patients is a non-random event, but is not associated with a parent-of-origin-dependent effect

Next, we undertook to determine whether the choice of an allele in the cancer-related process of allele-specific replication is a random event. Using the informative (heterozygous) *CEN15 *samples, we analyzed the SD-cell population for *CEN15 *and found that the early replicating *CEN15 *allele in the cancer samples is not selected at random. Specifically, in three out of the six cancer patients (Y, B, and S), the early replicating allele was the large one, showing the D-signal in 65% or more of SD cells for *CEN15*. In the other three cases (J, A, and C), the early replicating allele appeared to be the small one, showing the D-signal in 60% or more of the SD-cells for *CEN15 *(Fig. 4d).

It is noteworthy that in the occasional SD-cell population for *CEN15 *(low SD frequency) that appeared in the samples of non-cancer individuals, the selection for an early or late replicating *CEN15 *allele appeared to occur at random. This was evident from the equal frequencies (50% each) of cells with an early replicating large allele and an early replicating small allele observed in the SD-cell population for *CEN15*, in each of the six informative samples of the non-cancer individuals (Fig. 4b). These small populations of SD cells (usually found in synchronously replicating regions) differ from the large SD-cell population characterizing asynchronous replication and most likely result from background noise.

Finally, we checked whether the non-random selection of an early- or late- replicating *CEN15 *allele in the cancer samples is a parent-of-origin-dependent process. Apparently it is not. Proof comes from the lack of coordination in the replication status between the *CEN15*-allele and the *SNRPN*-allele assigned to the same homologue. The early replicating *CEN15 *allele in cancer cases A and C resided on the same homologous chromosome as the early replicating *SNRPN *allele. On the other hand, in cancer cases Y and J, the early replicating *CEN15 *and *SNRPN *alleles were assigned to different homologues (bars Y, J, A, and C in Fig. 4c and 4d). This indicates that the cancer-related asynchronous replication in the *CEN15 *region, while clearly not random, is guided by a mechanism other than a parent-of-origin-dependent one.

## Discussion

The asynchronous pattern of replication of the *SNRPN*-imprinted locus, consisting of early replication of the paternal allele (exemplified here in cells of the healthy son (K2) of a non-cancer patient) is in accord with the large amount of data documenting an allele-specific parent-of-origin replication mode for this locus in normal human cells [20-24]. The *SNRPN *pattern of replication differs significantly from the normal synchronous pattern observed for biallelically expressed genes [19,25,26,35,39], shown here in cells of the non-cancer patients by a non-transcribed locus – the chromosome-15 pericentromeric DNA array (*CEN15*).

However, in PHA-stimulated peripheral blood lymphocytes of the cancer patients, the *SNRPN *gene exhibited a relaxation, almost a loss, leaving only remnants of its characteristic asynchronous pattern of replication. This confirmed our earlier finding that blood cells of patients with urological cancers display relaxation in the asynchronous pattern of allelic replication characterizing imprinted loci [25,26]. It is noteworthy that loss of asynchronous replication of *SNRPN *was used to confirm lack of imprinting in cells of individuals carrying uniparental disomy for the *SNRPN *locus [21,23,24,40]. In addition to loss of the asynchronous replication of the imprinted locus, the blood lymphocytes of the cancer patients studied here exhibited extreme changes in replication timing of the *CEN15 *locus, which was asynchronous. These findings confirm our previous findings of cancer-related, non-locus specific replication timing alterations of loci (that normally replicate synchronously) in blood lymphocytes of patients with a solid tumor (see Introduction). Thus, an aberrant epigenetic profile is seen in blood cells of cancer patients, one marked by non-locus specific asynchronous replication of normally biallelically expressed loci and synchronous replication of imprinted genes.

Yet, the aberrant epigenetic profile in blood cells of the cancer patients was reverted to normal in the presence of AZA, a classical methylation blocking agent, linking the global cancer-related replication timing alterations to methylation capacity (discussed later). These results are in accord with findings that an AZA analog, 5-aza-2'-deoxycytidine, which mimics AZA in its demethylation activities, restored the normal imprinting in cancer cells exhibiting LOI [41,42]. Here, we show that AZA reinstated the asynchronous replication of *SNRPN *in cells of the cancer patients by re-establishing the advanced replication of the normally early replicating (paternal) allele. This suggests that the cancer-related loss of asynchronous replication of *SNRPN *observed here resulted from delayed replication (due to cancer-mediated hypermethylation) of the normally early replicating paternal allele, rather than advanced replication of the normally late replicating maternal allele. It cannot be ruled out, however, that the loss of *SNRPN *asynchronous replication resulted from advanced replication of the late replicating (maternal) allele. In fact, classical reports of LOI documented that cancer related relaxation of imprinting may arise either from activation of the normally silent allele or from inactivation of the normally expressed allele [36,43].

*CEN15*-replication behaviour, in normal situations as well as in reaction to cancer status and to the methylation blocking agent, completely mimics a biallelically expressed locus. This is shown here by the similarity between the *CEN15 *and the retinoblastoma gene *RB1*: the first known tumor suppressor gene, also the first to show an epigenetic inactivation – hypermethylation – rather than a genetic inactivation in a tumor suppressor gene, and to disclose that this inactivation was linked to an allele-specific event [38]. It is therefore reasonable to assume that *CEN15 *fully characterizes biallelically expressed loci; and, assuming that *SNRPN *reliably represents an imprinted locus, it appears that LOI is only one aspect of a much broader cancer-related epigenetic alteration, namely, loss of the inherent coordination between alleles. This alteration is neither locus- nor disease-specific, and is erased in the presence of AZA-like methylation-blocking agents. The alteration was evidenced here by loss of replication timing properties of three unrelated loci in blood cells of prostate cancer patients.

The FISH replication assay as used here (avoiding S-phase cells labeling) is a simple and reliable method for replication timing analysis. First, we show here that in the normal samples, it repeats results obtained by others in labeled S-phase cells [17,18,20-24]. Second, it is evident that the considerably low frequency of SD cells for *SNRPN *characterizing the cancer cases cannot be attributed to shortening of the duration of the S-phase since at the same time these same samples revealed a significant increase in the frequency of SD cells for *CEN15 *and *RB1*. Similarly, while an increase in the S-phase duration may explain the increase in the frequency of SD cells for *CEN15 *and *RB1*, it fails to explain the dramatic decrease in the frequency of SD observed for the *SNRPN *locus. Furthermore, because FISH uses single cells rather than bulks of DNA, it appears to be especially sensitive for differentiating between the synchronous and asynchronous modes of allelic replication. In addition, the assay estimates only stages prior to or after termination of the whole replication process of each counterpart of a tested locus, rather than pooled S-phase cells, some of which are trapped in the course of the replication process of the tested locus [15,17,18].

Using cell samples of cancer patients that are heterozygous for a size polymorphism at the *CEN15 *region enabled us to show that the selection of an allele for early or late replicating in the cancer-related asynchronous replication was not random; in each sample, most of the informative cells revealed one specific allele exhibiting early replication, either the small or the large one. This non-random choice was not dependent on the parental origin of *CEN15*, because in some cases the early replicating *CEN15 *allele was assigned to the same chromosome on which the early replicating *SNRPN *locus resided (paternal chromosome), and in others on the chromosome carrying the late replicating *SNRPN *locus (maternal chromosome). This suggests that the selection of a *CEN15 *allele for allele-specific replication in the cells of cancer patients is similar to the choice of an early (active) or late (inactive) replicating chromosome in the process of X-inactivation [44,45]; furthermore, it resembles the selection of the early (active) allele in the process of allelic exclusion [11,15]. Neither mechanism is parent-of-origin-dependent, but both (one made on the chromosome level and the other on the allelic level) ensure that the selected homolog (allele) for early (or late) replication passes the information from one generation to the next in a cell lineage. However, the mechanism that renders the two copies of a locus different from one another is unknown.

It was proposed that a normally existing variation in the accumulation of long interspersed nuclear elements (LINE)-1 facilitates differentiation between homologous counterparts for X-inactivation [46], as well as for the allele-specific expression of autosomal genes [47]. Since considerably high densities of LINE-1 repeats also appear in various autosomal regions not known to exhibit allelic differences in gene expression, the role of LINE-1 repeats in the mechanism initiating allelic functional asymmetry seemed doubtful [47]. However, recent data showing that monoallelically expressed genes are more widespread on autosomes than expected [48] may strengthen the LINE hypothesis, raising the possibility that the cancer status takes advantage of such a variation. Furthermore, it seems that those recently discovered autosomal monoallelically expressed genes retain an inherent functional plasticity with regard to monoallelical and biallelical expression [2,48], which may facilitate the shifting from one mode of expression to the other in response to malignancy.

Different nuclear positions of alleles in the interphase nucleus at the S-phase were reported to accompany allelic asymmetry [10,12,18]. Hence, one may speculate that the epigenetic disequilibrium linked to the cancer phenotype [36] alters the spatial DNA organization within the nucleus, positioning each of the parental sets at a different replication domain and thereby affecting the epigenetic symmetry of various genes at once.

The mechanism involved in maintenance of allele-specific replication, observed here in response to cancer, may resemble that involved in maintaining the functional asymmetry between the two homologous counterparts resulting from both X-inactivation and allelic exclusion. Both these processes are maintained by the methylation capacity of the genome [11,49], similar to what we observed in the cancer-related asynchronous replication. However, the asynchronous replication acquired by the cancer-status, probably later in life (shown here for *CEN15 *and *RB1 *in cells of cancer patients), differs in the response to AZA from that hatched normally into genes at early developmental stages (exemplified here by *SNRPN *in cells of non-cancer patients). We show here that the former is reversed in the presence of AZA, and the latter is unaffected. Our findings are in accord with reports claiming that AZA-like drugs activate genes subjected to epigenetic silencing, particularly if the silencing occurred due to a pathological situation, making these drugs efficacious in treating cancer [50-52]. Specifically, AZA and its analogs are active only in S-phase cells, as they became incorporated (in place of cytosine) into replicating DNA. The newly formed azacytosine-containing DNA blocks methyltransferases activity [50]. As such, these drugs generates heritably demethylated DNA, and, thus, activate silent genes, shifts replication timing of various DNA sequences to early replication S-phase domains [53].

According to our results, each of the cancer patients had an allele-specific replication mode for loci that normally replicate in the classical biallelic-mode. Each of the non-cancer patients showed the expected synchronous patterns of replication for each of these loci. Taken together, these results suggest that the aberrant replication mode is a response to the disease and not an inborn- or age-acquired cancer-predisposing epigenetic marker. This holds true also for the aberrant replication shown for the imprinted locus. This concept is strengthened by the fact that the non-cancer patients tested here were at an advanced age and therefore at increased risk for developing cancer compared to the normal population. Hence, the allele-specific labelling, in contrast to X-inactivation and allelic exclusion, most probably occurs during the lifespan of an individual rather than at the prenatal early developmental stages. This accords with the idea that epigenetic alterations associated with modifications in methylation capacity may take place later in life as well [50,54]. However, it should be emphasized that the cancer-based aberrant patterns of replication are not age dependent, as they characterize young cancer patients as well as elderly ones [35].

Finally, a cancer-related, non-disease specific, aberration observed for a large number of loci in a single cell sample, achieved by low invasive means, offers a way to identify potential epigenetic biomarkers for cancer detection and disease follow up. Besides, an aberration that can be reversed to normal by an epigenetic drug applied in vitro may provide a candidate marker for cancer drug evaluation.

## Conclusion

The cancer-related, non-locus specific, allelic replication timing aberration, observed in peripheral blood cells, is linked to aberrant methylation and may be reversed to normal by an epigenetic drug applied in vitro. It is analogous, although broader in manifestation, to loss of imprinting, a phenomenon widely associated with malignancy. The choice of an allele for early or late replication in the cancer-related asynchronous replication is not random but independent of the parental origin.

## Competing interests

The authors declare that they have no competing interests.

## Authors' contributions

ZAD participated in the design of the study, carried out most of the cytogenetic studies, performed the statistical analyses and helped to draft the manuscript. AD participated in the design of the study, carried out a part of the cytogenetic studies, and helped to draft the manuscript. JR participated in the design of the study. LA conceived the study, and participated in its design and coordination and drafted the manuscript. All authors read and approved the final manuscript.

## Pre-publication history

The pre-publication history for this paper can be accessed here:


